# Three-dimensional electro-neural interfaces electroplated on subretinal prostheses

**DOI:** 10.1088/1741-2552/ad2a37

**Published:** 2024-02-23

**Authors:** Emma Butt, Bing-Yi Wang, Andrew Shin, Zhijie Charles Chen, Mohajeet Bhuckory, Sarthak Shah, Ludwig Galambos, Theodore Kamins, Daniel Palanker, Keith Mathieson

**Affiliations:** 1 Institute of Photonics, Department of Physics, University of Strathclyde, Glasgow, United Kingdom; 2 Hansen Experimental Physics Laboratory, Stanford University, Stanford, CA, United States of America; 3 Department of Physics, Stanford University, Stanford, CA, United States of America; 4 Department of Materials Science and Engineering, Stanford University, Stanford, CA, United States of America; 5 Department of Electrical Engineering, Stanford University, Stanford, CA, United States of America; 6 Department of Ophthalmology, Stanford University, Stanford, CA, United States of America

**Keywords:** retinal degeneration, three-dimensional electrode, honeycomb electrode, pillar electrode, retinal prosthesis

## Abstract

*Objective.* Retinal prosthetics offer partial restoration of sight to patients blinded by retinal degenerative diseases through electrical stimulation of the remaining neurons. Decreasing the pixel size enables increasing prosthetic visual acuity, as demonstrated in animal models of retinal degeneration. However, scaling down the size of planar pixels is limited by the reduced penetration depth of the electric field in tissue. We investigated 3-dimensional (3d) structures on top of photovoltaic arrays for enhanced penetration of the electric field, permitting higher resolution implants. *Approach.* 3D COMSOL models of subretinal photovoltaic arrays were developed to accurately quantify the electrodynamics during stimulation and verified through comparison to flat photovoltaic arrays. Models were applied to optimize the design of 3D electrode structures (pillars and honeycombs). Return electrodes on honeycomb walls vertically align the electric field with bipolar cells for optimal stimulation. Pillars elevate the active electrode, thus improving proximity to target neurons. The optimized 3D structures were electroplated onto existing flat subretinal prostheses. *Main results.* Simulations demonstrate that despite exposed conductive sidewalls, charge mostly flows via high-capacitance sputtered iridium oxide films topping the 3D structures. The 24 *μ*m height of honeycomb structures was optimized for integration with the inner nuclear layer cells in the rat retina, whilst 35 *μ*m tall pillars were optimized for penetrating the debris layer in human patients. Implantation of released 3D arrays demonstrates mechanical robustness, with histology demonstrating successful integration of 3D structures with the rat retina *in-vivo*. *Significance*. Electroplated 3D honeycomb structures produce vertically oriented electric fields, providing low stimulation thresholds, high spatial resolution, and high contrast for pixel sizes down to 20 *μ*m. Pillar electrodes offer an alternative for extending past the debris layer. Electroplating of 3D structures is compatible with the fabrication process of flat photovoltaic arrays, enabling much more efficient retinal stimulation.

## 1. Introduction

Age-related macular degeneration (AMD) is one of the leading causes of irreversible sight loss worldwide [1], affecting an estimated 200 million people. In its atrophic form, called geographic atropy (GA), this degenerative retinal condition leads to loss of the photoreceptor cells in the central macula [2], the high resolution region of the retina responsible for our central vision, thus impairing patients’ ability to read and recognize faces. Despite the loss of photoreceptors, the inner retinal neurons can remain functional, and electrical stimulation of these neurons can evoke visual percepts [3]. Recent clinical trials with a subretinal photovoltaic array PRIMA (Pixium Vision, Paris, France) demonstrated form perception in GA of AMD patients, with prosthetic acuity reaching the level of 20/438, closely matching the implant’s pixel size of 100 *μ*m, which corresponds to the acuity limit of 20/420 [4]. Since the remaining peripheral vision in AMD patients often supports visual acuity of no worse than 20/400, clinically meaningful improvement requires smaller pixels. For example, a visual acuity exceeding 20/100 would require pixels of about 20 *μ*m [5]. Patterned electrical stimulation of the retina with 20 *μ*m pixels has demonstrated a grating acuity up to the natural resolution limit of 27 *μ*m in rats [6]. However, new strategies are needed to safely translate this to a significantly thicker human retina [7].

Subretinal implants aim to activate the bipolar cells (BC) in the inner nuclear layer (INL) [3] by polarizing them in electric field, and then rely on the remaining retinal neural network to process their output and evoke the bursts of action potentials in the retinal ganglion cells. Utilizing this remaining retinal network has been shown to preserve many features of the retinal signal processing, including flicker fusion, antagonistic center-surround, and others [8].

In the PRIMA system, the near-IR pulses (880 nm) projected onto the photovoltaic implant from the augmented-reality glasses are converted into pulses of electric current, injected into electrolyte via the active electrodes in each pixel and collected by the return electrodes surrounding each pixel. Decreasing the pixel size can increase the achievable visual acuity, but stimulation thresholds rapidly increase [9] due to reduced penetration of E-field into the tissue and reduced photosensitive area in each pixel. They can be compensated by higher IR irradiance, but for pixels smaller than 40 *μ*m in rodents and 75 *μ*m in humans, the required irradiance exceeds the ocular safety limit for near-IR exposure (8.25 mW mm^−2^ at 10 ms pulse duration and 30 Hz repetition rate) [10]. Stronger stimuli are required with human retina because it is thicker than in rodents and because it exhibits a 35 *μ*m subretinal debris layer in atrophic areas, which increases the separation between the target cells and the implant [11].

3D electrode structures offer a solution to this problem, as the stimulating electric field can either be shaped for more efficient stimulation or brought closer to the target neurons. Previous studies with passive 3D implants demonstrated that inner retinal neurons migrate into the voids in the implant, and thereby can achieve close proximity to electrodes [9, 12–14]. Two types of 3D electrode structures have been proposed: a raised return electrode in a hexagonal array (so-called honeycombs) [9] and pillar electrodes that raise the active electrode to the target neuronal layer [12]. Both approaches have advantages and limitations. For example, the honeycomb structures align the electric field vertically within the well, matching the dominant orientation of BCs, thus reducing their stimulation threshold and decreasing the pixel-to-pixel cross-talk. However, it is unclear how such structures will integrate with a debris layer in human retina. Pillar electrodes, on the other hand, may penetrate through this debris layer, bringing the stimulation site close to the target inner retinal neurons. However, the spread of current from the pillar top is more spherical, so that the threshold and contrast may be degraded, compared to honeycombs. Previously, we investigated short (10 *μ*m) pillars in Royal College of surgeons (RCS) rats, where there is no subretinal debris, and observed a moderate (2-fold) reduction in stimulation threshold with 55 *μ*m pixels [15]. The pixels investigated here are much smaller—down to 20 *μ*m, and pillars are much taller (35 *μ*m in height), shown in supplementary figure 1, designed to raise the active electrode above the debris layer between implant and the INL in humans [16], and thus a much more significant reduction of the stimulation threshold is expected.

**Figure 1.**
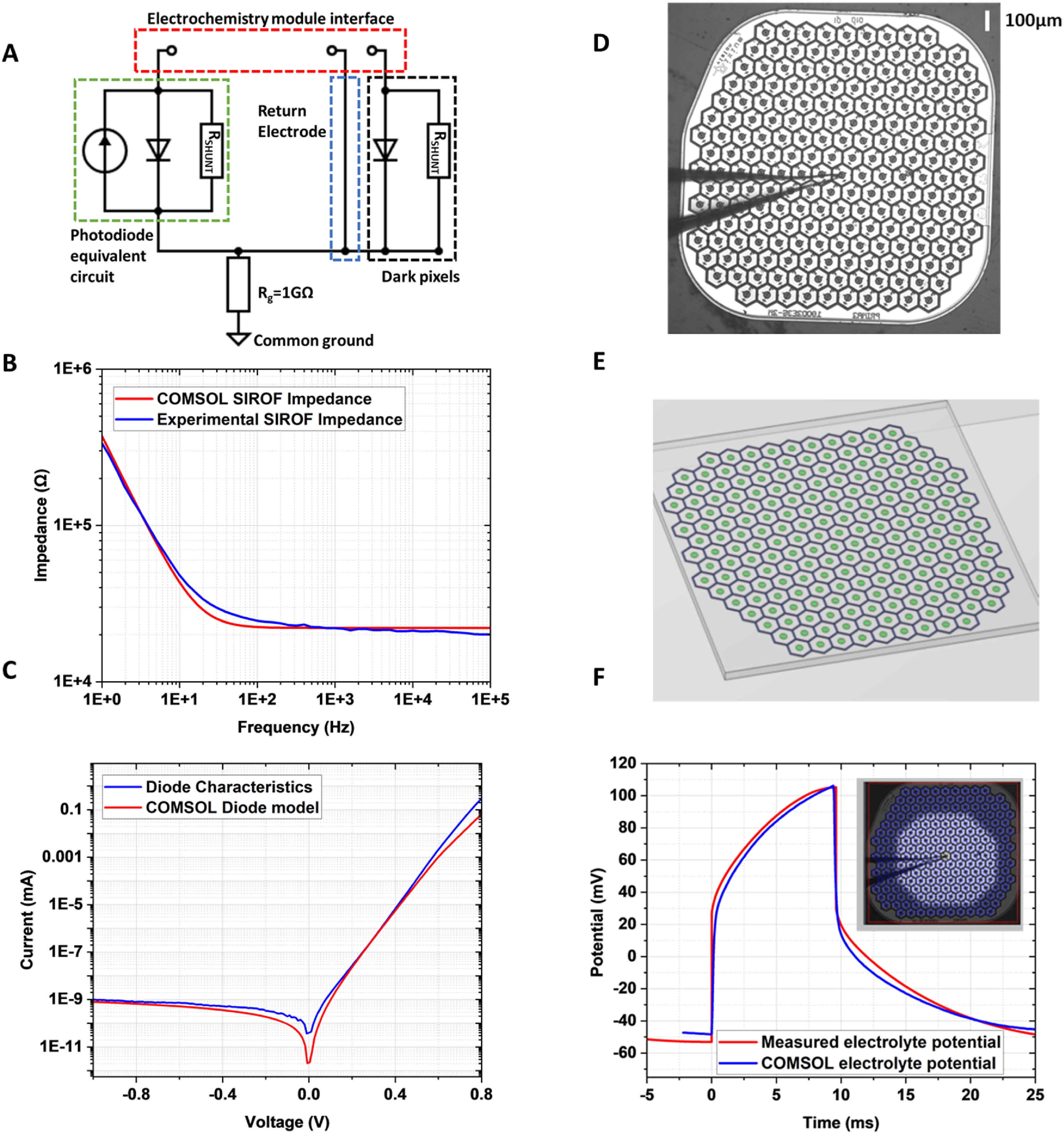
(A) SPICE circuit model used to drive the input electrical signals into the COMSOL electrochemistry model. Each modeled pixel is represented by an equivalent circuit model coupled to the stimulation (active) electrode in the electrochemistry solution, where the hexagonal return electrodes are connected through a common terminal. Illuminated pixels also have a current source. (B) Modeled and experimental measurements of electrode impedance across frequencies for a 0.1 V pk-to-pk sinusoidal input to an 80 *μ*m diameter active SIROF. (C) I–V characteristics of a diode in the SPICE circuit model compared to experimental results from fabricated photodiode array [4]. (D) Experimental setup for electrolyte potential measurement. A PRIMA implant was submerged in NaCl solution (1.52 mS cm^−1^), and a micro-pipette electrode used to measure electrolyte potential at 17 *μ*m above the device. (E) This experimental setup was replicated in the electrochemistry model in COMSOL. (F) Experimental and modeled electric potential 17 *μ*m above the implant under spot illumination (diameter = 1000 *μ*m, λ = 880 nm, irradiance = 3 mW mm^−2^, pulse duration 10 ms, rep. rate 30 Hz).

These high-aspect ratio structures present a fabrication challenge, and we describe electroplating process for such 3D electrodes on a photovoltaic implant. To assess their expected performance, the structures are modeled using 3D finite element analysis (COMSOL Multiphysics 5.6 with electrochemistry and circuit modules). This model was first verified by comparison with experimental results from a Pixium PRIMA chip (figure 1(D)). It was then extended to model an array of conductive 3D structures acting as return electrodes on honeycombs or active electrodes on pillars. This model informed the fabrication process for both of these devices, highlighting the effect of the low-capacitance side walls and the high-capacitance top coating of the 3D structures. The developed fabrication process is compatible with the existing design of the photovoltaic retinal implants, and thus immediately translatable into *in-vivo* testing.

## 2. Methods

### 2.1. Modeling

The finite element analysis tool, COMSOL Multiphysics, was used to calculate the potential throughout the modeled conductive domain. Analysis was carried out using the electrochemistry module in three dimensions to simulate the electrolyte regions and the electrode-electrolyte surface boundaries. Electric potential was computed by coupling the Poisson equation for current density in the electrolyte with the Nernst-Planck equation for flux of charge carriers, assuming electroneutrality and negligible charge carrier gradients [17]. Anodic and cathodic reactions at electrode surfaces were modeled using the Butler–Volmer equation. The electrochemistry module was coupled to a circuit model in COMSOL, which represented individual photodiodes, driving current to active electrodes in illuminated pixels, as well as a path to the interconnected return electrodes. Using these coupled models, allows simulation of the access resistance, double layer capacitance, electrode kinetics and electrolyte potential through the electrochemistry module, whilst the circuit model can drive the stimulation currents and allow electrode surfaces to have floating potentials, which change over time [18]. Using this approach, current is injected into the electrochemical system, as defined by the circuit model, and is collected by either the return electrodes, or the adjacent active (stimulation) electrodes, the potential of which is determined by the circuit dynamics (figure 1(A)). A 1 mm diameter ring electrode is placed 1 mm above the modeled array, defined as 0 V and connected to the common ground in the circuit model analysis.

Potentials are in general referenced to this distant electrode. However, for stimulation of BCs, the important metric is the potential drop across the cell layer [19], and so we reference stimulation potentials to the point where the axons of the BCs terminate (middle of IPL at 57 *μ*m above the implant).

### 2.2. Model verification

To calibrate the model we compared: (1) modeled electrode impedance values to electrochemical impedance spectroscopy (EIS) measurements from microelectrode structures [18]; (2) the current-voltage characteristics for the modeled photodiode to the experimental results from our photovoltaic device [4] and (3) the computed electric potential to the measured voltage pulses [7] in electrolyte generated by PRIMA implants.

The impedance of a SIROF (sputtered iridium oxide film) electrode surface was modeled using the EIS component of the electrochemistry module in COMSOL. Based on our previous measurements, the SIROF capacitance was set to *C*_Sirof_ = 8.52 mF cm^−2^, for a sodium hypochlorite cleaned surface [18]. SIROF is used for the active electrode and return electrodes due to its high charge injection capacity (CIC) compared to other electrode materials [20]. Even though reversible Faradaic reactions contribute to the high capacitance of SIROF, known as the pseudo-capacitance, we combine the double-layer and faradaic capacitances as C_DL_ in COMSOL. To enable a direct comparison with the previously published experimental data [18], we set the conductivity of the electrolyte domain to 2.83 mS cm^−1^ to match the dilute phosphate buffered saline solution used in these experiments. An exchange current density of 1 mA cm^−2^ was set for the SIROF electrode interface [21]. A frequency sweep was performed and plots of the absolute value of impedance against frequency showed close agreement with the experimental results—figure 1(B).

As shown in figure 1(A), the pixel equivalent circuit is modeled as a current source, a diode and a shunt resistor in parallel. The *I*–*V* characteristics of the diodes used in this equivalent circuit were set to match the photodiodes of the retinal prosthesis detailed in [4]: junction capacitance of 30 pF, ideality factor of 1.5, responsivity of 0.51 A W^−1^ (figure 1(C)).

### 2.3. 3D electrode model

With the circuit model and electrode/electrolyte interfaces set, we modeled an array of 100 *μ*m pixels, matching a PRIMA implant—figures 1(D) and (E), and evaluated the electrolyte potential 17 *μ*m above the device. This was compared to experimental recordings via pipette electrode positioned 17 *μ*m above the PRIMA array in a diluted saline solution [7] and illuminated at 3 mW mm^−2^ with pulses of 9.6 ms in duration at 30 Hz repetition rate. Note that conductivity was changed to 1.52 mS cm^−1^ to match the experimental value in [7]. The voltage transients were measured with respect to a large Ag/AgCl wire in a Petri dish, which served as a ground electrode. As shown in figure 1(F), the simulated output closely matches the experimental waveform, demonstrating that the model accurately represents the photovoltaic arrays in electrolyte.

To model the 3D honeycomb arrays, 25 *μ*m tall walls of 4 *μ*m width were added on top of the pixel return electrodes with a 22 *μ*m pitch. Each pixel contained a central active electrode, 9 *μ*m in diameter and 400 nm in height. These 3D structures were positioned on a 30 *μ*m thick substrate, which represents the silicon photovoltaic implant, and placed within a 150 *μ*m thick layer (conductivity 1 mS cm^−1^ [9]) to represent the retina, within a 1 mm cube representing the vitreous (conductivity 15 mS cm^−1^ [22]). A 500 *μ*m inner radius and 20 *μ*m diameter ring electrode surrounded the modeled array to act as an additional distant return electrode. The honeycomb walls were modeled as gold, while the active electrodes and caps on top of the walls, modeled as 400 nm thick SIROF. Current pulses are defined in the circuit model, which then determines the current and voltage on active and return electrode interfaces in the electrochemisty module. All other surfaces are defined as electrically insulating (Neumann boundary conditions). Due to the shunt resistors and the diode conductivity under sufficient bias, the active electrodes in non-illuminated pixels (both honeycomb and pillar models) can collect current just like the return electrode mesh in the honeycomb model. Pillar active electrodes were modeled by placing the 9 *μ*m diameter SIROF active electrode on top of a 35 *μ*m high Au pillar (same diameter) and using the 0.5 mm radius ring as a common return electrode.

The magnitude of the current source in each pixel was calculated based on illumination of 1 mW mm^−2^, taking into account the photosensitive area of a pixel and responsivity of 0.5 A W^−1^ at *λ* = 880 nm wavelength [4]. A shunt resistor is included in each pixel to help discharge the electrode capacitors between the light pulses (30 Hz, 4 ms pulse width). The optimal value of the shunt resistor depends on the pixel size. Using a value of approximately five times the access resistance, a shunt of 720 kΩ was selected for 100 *μ*m pixels. When modeling the 20 *μ*m pixel arrays, a shunt value of 4 MΩ was selected using the same criteria. The side walls (C_DL_ = 14–100 *μ*F cm^−2^) and SIROF caps of the return electrodes in each pixel are connected to the terminals of the circuit model, and all the return electrodes are connected together in one common mesh. All current applied through the stimulation (active) electrode is collected by the other electrode surfaces, such that the total charge in the system is conserved.

### 2.4. Fabrication of 3D electrodes

We have previously described the fabrication process for planar photovoltaic retinal implants [4]. Here we detail fabrication processes and procedures for integration of the 3D electrode structures, building upon established fabrication procedures of the planar devices. These electrodes are electroplated onto the photovoltaic arrays after the fabrication of photodiodes, but before the electrode interface material (SIROF) is deposited.

In order to develop this process on a protoype wafer, we patterned the active and return electrode structures in a Ti:Au layer (50 nm:200 nm) on a blank 4 inch silicon wafer (p-doped) using a lift-off process (500 nm layer of LOR-10B, followed by a layer of Shipley 1805 photoresist). These active and return electrode structures, used as starting points for electroplating the 3D devices, were interconnected across the entire wafer, allowing simultaneous electroplating. Releasable arrays were fabricated by etching a 30 *μ*m trench (also 30 *μ*m wide) using a deep reactive ion etching process into a blank 4-inch silicon wafer (p-doped). A narrow Si bridge (measuring 2.3 *μ*m at the narrowest point mid-way across the trench) was left in each array to connect the implant area to the bulk Si wafer. This proves a support for an electric contact from the edge of the wafer to the electroplating area of each implant. The narrow bridge cleaves at the narrow point after device release. After that, a 300 nm layer of platinum was coated across the wafer to provide electroplating connections. Dimensions of the electroplated structures were chosen to match those used in photovoltaic subretinal prostheses, where each hexagonal pixel consisted of a disc electrode in the middle and a circumferential electrode on the edge [4]. Each array was 1.5 mm in diameter, comprised of pixels of 55, 40, 30 or 22 *μ*m in width. For honeycombs, the circumferential electrode of each pixel was electroplated into vertical walls of widths 5.5 *μ*m (55 *μ*m pixels), 4.5 *μ*m (40 *μ*m pixels) and 4 *μ*m (30 and 22 *μ*m pixels). For the pillar design, the disk electrode in every pixel was electroplated into a pillar, with diameters of 22, 16.5, 11.5 and 8.5 *μ*m for pixels of 55, 40, 30 or 22 *μ*m, respectively.

A thick high-aspect ratio negative photoresist (KMPR-1025) was used to define the mask for electroplating gold honeycombs or pillars. The electroplating pattern was transferred using a contact aligner (Karl Suss MA6), and development was carried out with a TMAH-based developer. Before electroplating, a descum process was carried out using a reactive ion etching tool to ensure the base of the photoresist pattern is effectively cleared. Patterned wafers were fixed into a custom-made, PTFE wafer holder, which isolated the back surface and edges of the wafer, so that only the desired areas were exposed to the gold electroplating solution (NB Semiplate AU 100^TH^, NB Technologies, Bremen, Germany). A hollow handle provided electrical contact to the Ti:Au layer on the wafer surface, while a platinized titanium mesh, positioned parallel to the wafer surface, was used as the anode. A hot plate kept the solution at a temperature of 30 °C and a stirrer provided constant agitation at 40 rpm. A constant current density of 1 mA cm^−2^ was applied, providing a plating rate of 3 *μ*m per hour. After electroplating up to the desired height, the solution was removed, and the wafer rinsed with DI water. The KMPR-1025 electroplating template was removed using PG remover at 80 °C, leaving the desired 3D honeycomb or pillar pattern in gold.

To coat the tops of the walls and pillars with a SIROF layer, a lift off process was used. Once electroplated, the wafers were spray-coated in photoresist (50 *μ*m, SPR-220-7) and processed through a repetitive cycle of underexposure and development to remove the resist, in a layer by layer fashion, until the top of the electroplated metal structures were revealed. The top surface of the electroplated structures was then sputter-coated with Ti:SIROF (40 nm:436 nm), providing a high-capacitance material for the electro-neural interface. The fabrication procedure concludes by dissolution of the remaining photoresist, revealing the 3D walls and pillars with SIROF on the top surface and exposed Au on side walls. The wafers were thinned from 450 *μ*m to 50 *μ*m using a commercial back-side grinding process (GDSI Inc. San Jose, CA). In preparation, the wafers were spray coated with a thick photoresist layer (70 *μ*m, SPR-220-7) to protect the front side of the wafer. GDSI Inc. mounted the wafers on a UV tape (resist side down) allowing the 400 *μ*m of the Si wafer to be removed using the grinding process. Further XeF_2_ etching in an Xactix e-1 vapor etch tool allowed precise removal of a further 20 *μ*m of Si, leaving a 30 *μ*m thick Si substrate. This exposed the etched trenches around each device, and subsequent dissolution of the thick photoresist layer in acetone lifted off the devices from the supporting UV tape. The process flow for this fabrication is shown in supplementary figure 2.

Honeycomb walls were fabricated to 25 *μ*m in height (the approximate thickness of the inner nuclear layer), while pillar height was set to 35 *μ*m, to match the debris layer thickness in AMD patients [16].

### 2.5. Animals, surgical procedures and tissue processing

All experimental procedures were approved by the Stanford administrative panel on laboratory animal care and conducted in accordance with the institutional guidelines and conformed to the statement for the use of animals in ophthalmic and vision research of the association for research in vision and ophthalmology. RCS rats were used as a model of photoreceptor degeneration. Total of *N* = 3 animals were implanted with pillar arrays after the age of P180 to ensure complete degeneration of the photoreceptors. As previously described [3], animals were anesthetized with a mixture of ketamine (75 mg kg^−1^) and xylazine (5 mg kg^−1^) injected subcutaneously. A 1.5 mm incision was made through the sclera and choroid 1 mm posterior to the limbus. The retina was detached with an injection of saline solution, and the implant was inserted into the subretinal space at least 3 mm away from the incision site. The conjunctiva was sutured with nylon 10-0, and topical antibiotic (bacitracin/polymyxin B) was applied on the eye postoperatively. The eyes were collected 8 d later and fixed in 4% paraformaldehyde. The retinal whole mount was stained with DAPI nuclear marker, imaged by LSM 880 confocal microscope (Zeiss LSM 880, Germany) and reconstructed using ImageJ (Fiji) and MATLAB 2021b (Mathworks, Inc., Natick, MA).

## 3. Results

### 3.1. Modeling the neural stimulation

After validating the electrochemical model by comparison with experimental results, as described in sections 2.2 and 2.3, we investigated the effect of three-dimensional structures on the electric field generated by 22 *μ*m pixels. An array of 59 pixels was modeled as described in section 2.3, and simulations carried out using planar, honeycomb and pillar geometries. A 4 ms stimulation pulse was applied to the current source in the circuit model (figure 1(A)), with an amplitude calculated for illumination of 1 mW mm^−2^, responsivity of 0.51 A W^−1^ [4] and the photoactive area of a 22 *μ*m photovoltaic pixel—217 *μ*m^2^. There is a range of double layer capacitance values for gold in the literature, depending on the surface smoothness and preparation. For our modeling, we have selected a *C*_DL_ = 56 *μ*F cm^−2^ from [23], but also explored the effect of lower (14 *μ*F cm^−2^) and higher (100 *μ*F cm^−2^) values of *C*_DL_. An exchange current density of 2 nA cm^−2^ was used for the gold in electroplated walls and pillars [24].

**Figure 2.**
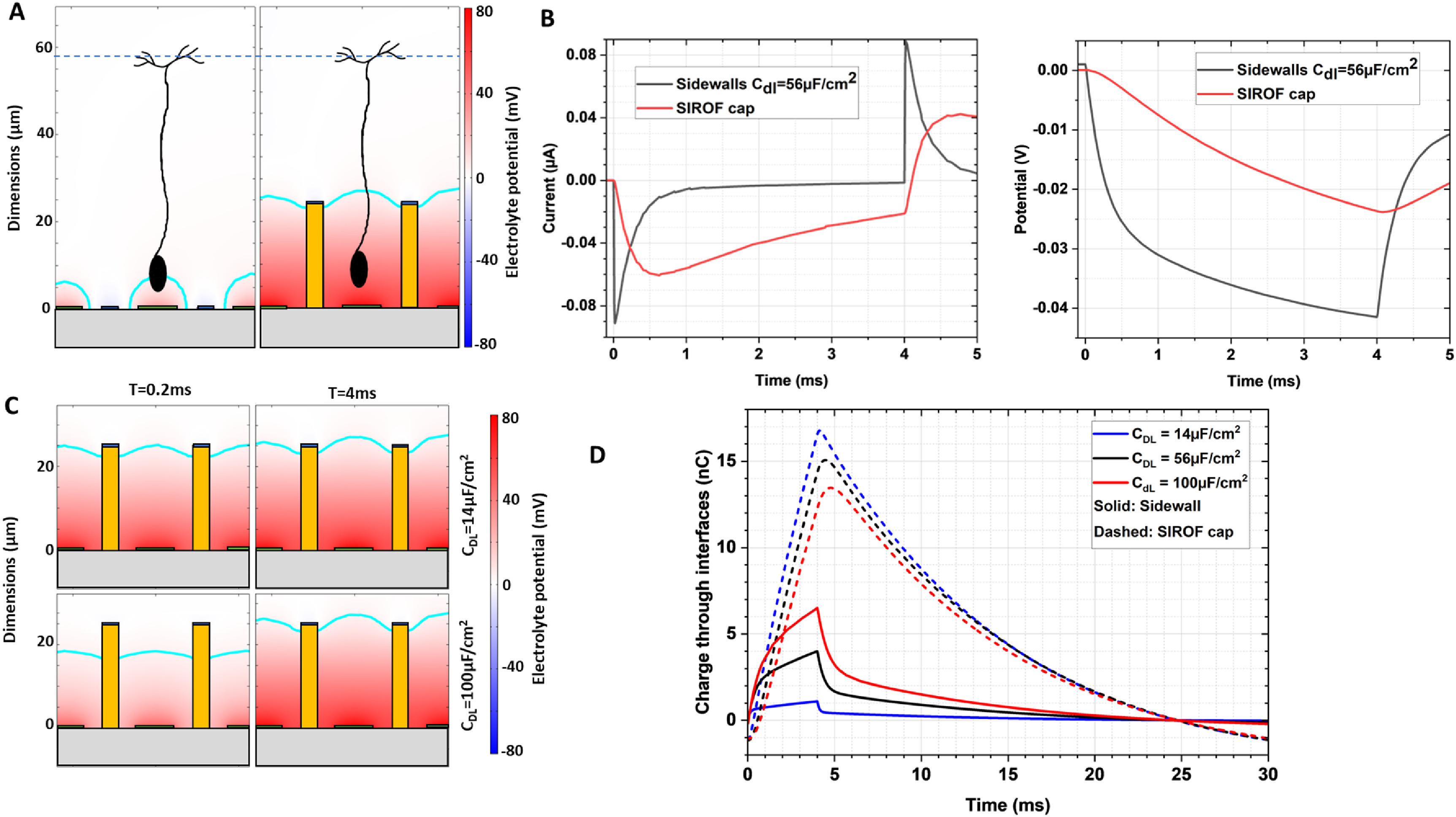
(A) Left: electric field penetration is limited for planar devices with small pixels, due to the proximity of the active and return electrodes. Right: placing the return electrodes on top of vertical walls, helps extend the field vertically and permits stimulation with smaller pixels. Electrolyte potential is depicted with respect to the middle of the IPL (57 *μ*m above the implant, shown in dash), where bipolar cell axons terminate. The cyan contour indicates the region above an assumed stimulation threshold of 4.3 mV. (B) Left: COMSOL model of the current flow across the return electrode structure. The electroplated sidewalls are modeled as electrical conductors, so initially, current flows into the sidewalls, but then the high capacitance SIROF coating that caps the return electrode structure, becomes the preferred current path. Right: The potential transient across the gold sidewall capacitive interface. In both panels in (B) red lines represent current and voltage for the SIROF cap whilst black lines represent these values for the side walls. (C) Different electrode materials exhibit a range of capacitances: two examples are shown for Au (14 *μ*F cm^−2^) and Pt (100 *μ*F cm^−2^) interfaces. Across this range of surface capacitances, current flow into the SIROF cap still dominates with little difference in the electrolyte potential profile at the end of a 4 ms pulse. (D) The total charge collected by the sidewalls and SIROF cap. With increasing sidewall capacitance, the charge collected by sidewalls (over a 4 ms pulse) increases, with a corresponding decrease in the charge collected through the SIROF cap.

Figure 2(A) depicts the electric potential in electrolyte in front of the planar and honeycomb arrays at the end of a 4 ms pulse. Diagram of a bipolar cell (BC) in front of the array (and migrated into the well) is shown to scale in this cross-section. The calculated electric potential in the retina is plotted with respect to the middle of the inner plexiform layer (IPL), 57 *μ*m above the surface of the array—the average location of BCs’ axonal terminals [9]. Previous work has shown that for a 4 ms anodic pulse, a potential difference of at least 4.3 mV across the BC, from soma to axonal terminals, is required to generate a retinal response [12]. This stimulation threshold is indicated in figure 2(A) as the cyan contour, demonstrating the field enhancement effect produced by the 3D structure. Within the honeycomb walls, this region extends towards the top of the wall, compared to a flat planar array, where it is confined to much smaller volume above the active electrode. Even though the three-dimensional return walls in our simulation are modeled as an electrically conducting surface, their small capacitance (14–100 *μ*F cm^−2^) results in this being a relatively high impedance path, compared to the SIROF cap electrodes (>4 mF cm^−2^). Figure 2(B) shows this effect in a model, where an initial spike of current flows into the sidewall at the beginning of a pulse, but then rapidly decreases as the sidewall capacitance charges up. Within 0.3 ms, majority of current starts flowing through the much higher capacitance of SIROF cap electrode, providing the vertical current alignment, matching the orientation of BCs. Notably, the cathodic potential transient on the Au sidewalls is approximately −40 mV during stimulation, well below the electrochemical reactions’ threshold for Au [23].

As mentioned above, there is a range of *C*_DL_ values for gold in literature, and we investigated the effect of changing *C*_DL_ from 14 to 100 *μ*F cm^−2^. Figure 2(C) shows electric potential with honeycombs at the onset and end of a 4 ms pulse. With a double layer capacitance set to 100 *μ*F cm^−2^ (bottom plots), the field penetration depth is initially restricted to ∼10–15 *μ*m. However, by the end of the pulse, current flows predominately through the SIROF cap, and electric field extends to the top of the walls. Figure 2(D) shows the time course of this process in terms of the charge collected by different surfaces. As the sidewall capacitance increases, the proportion of total charge collected by the sidewall also increases, from 6% for *C*_DL_ = 14*μ*F cm^−2^, 27% for *C*_DL_ = 56*μ*F cm^−2^, to nearly 50% for *C*_DL_ = 100*μ*F cm^−2^.

**Figure 3.**
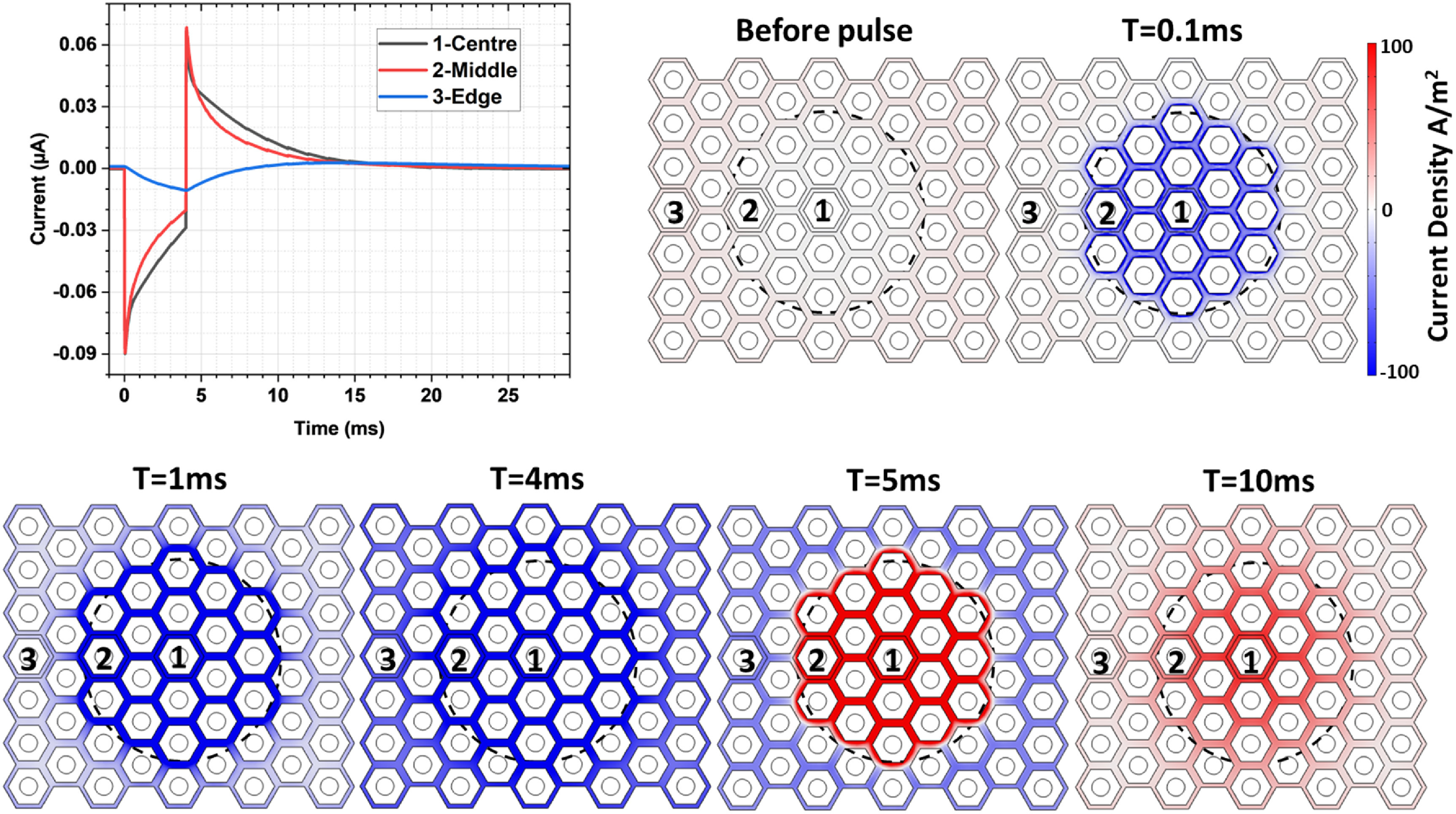
When a spot (indicated by the dashed circle) is illuminated by a 4 ms pulse, current is initially collected by the adjacent return electrodes, but over time it redistributes across the entire return electrode mesh. Similar redistribution occurs after the light pulse, with the return interfaces trending back towards equilibrium. Current density on active electrodes is not shown here.

The model also demonstrates the effect of charge redistribution across the return electrode surface. When 19 pixels are illuminated, as shown by dashed circle in figure 3, the current density on the top surface (SIROF) is initially confined to the return electrode surface within illuminated area. From 1 ms onwards, current begins to redistribute more evenly, recruiting the return electrode surfaces of non-illuminated pixels. This also occurs with the current returning in opposite polarity after the light pulse (*t* = 5 and 10 ms). This dynamic is summarized in a plot for electrodes at the center pixel (1), at the edge of illuminated circle (2) and in the non-illuminated area (3).

**Figure 4.**
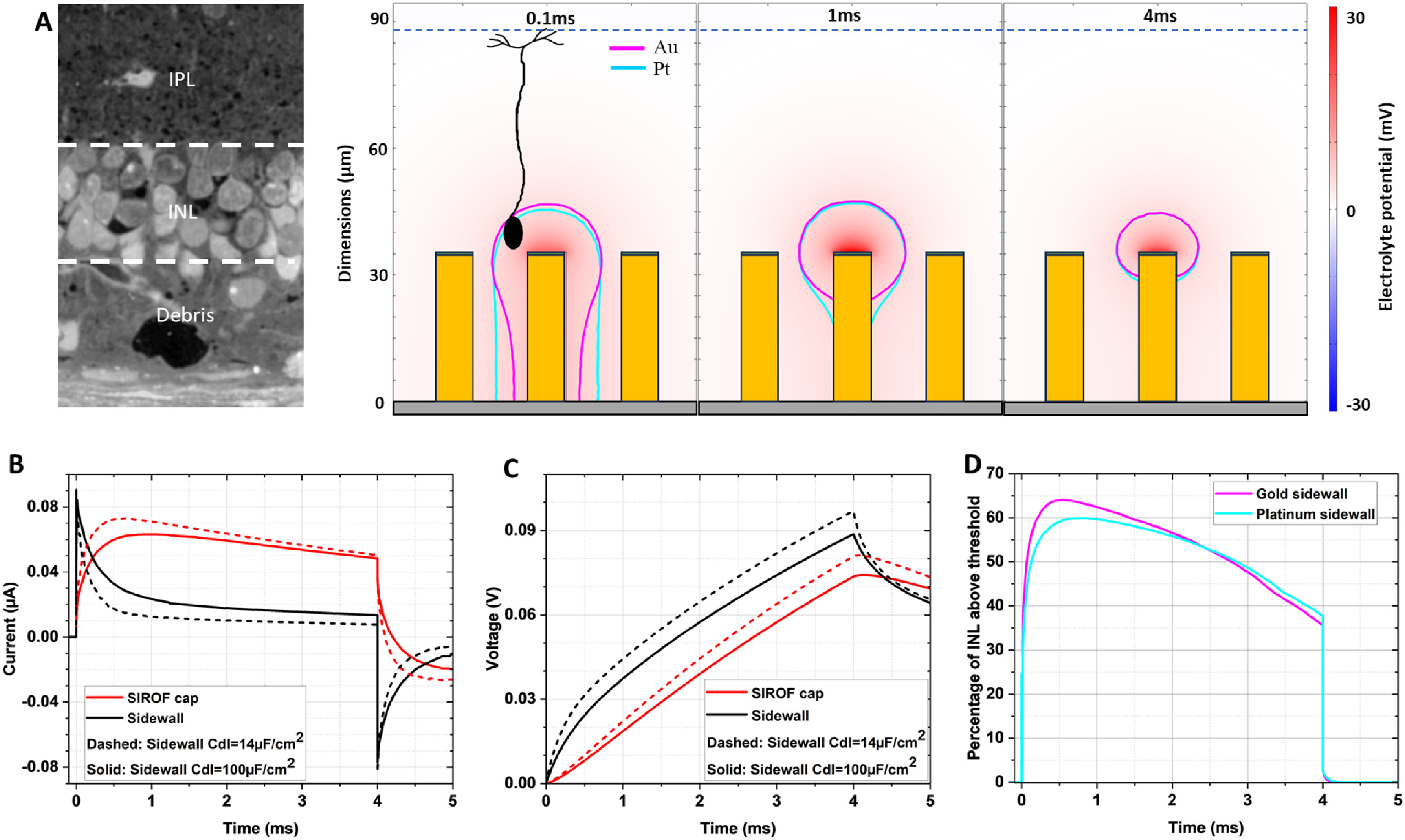
(A) The degenerated retina can exhibit debris layers up to 40 *μ*m thick in human patients. Electroplated pillars can penetrate this layer to deliver current to the targeted bipolar cells. The color map indicates the electrolyte potential with respect to the middle of the IPL for a gold pillar, and the purple contour indicates the volume above a stimulation threshold of 4.3 mV. Cyan contour indicates the same volume for a platinum pillar. (B) Stimulation through the pillar electrode, initially results in current flow out of the sidewall. However, this interface quickly saturates, and the preferred current path is then via the SIROF cap, where the majority of current is injected. (C) Potentials on the sidewall and SIROF cap during a 4 ms pulse. (D) Percentage of INL volume above the stimulation threshold of 4.3 mV during a 4 ms pulse. Previous work [9] has seen that VEP threshold corresponds to 8.3% of the INL volume above the stimulation threshold.

With 35 *μ*m tall, electroplated Au pillars acting as active electrodes, adjacent non-illuminated pixels can act as returns, collecting the injected current through the active electrodes via shunt resistor or via diodes under sufficient bias. With pillars, field is not shaped vertically as in honeycombs, but rather exhibits a spherical expansion, similar to the disc electrodes. Figure 4(A) shows the debris layer under the INL, which pillars are expected to penetrate, and evolution of the threshold potential difference (4.3 mV with respect to the middle of IPL) during the 4 ms pulse modeled using both gold and platinum (*C*_DL_ = 100 *μ*F cm^−2^) pillars. After initial charging of the side walls, the threshold contour becomes (and stays for the remainder of the pulse) localized to the top of the pillar, surrounding the SIROF cap. The current and voltage pulses are shown in figures 4(B) and (C). Notably, the pillar sidewall potential does not exceed 100 mV vs the distant reference electrode, well below the threshold of oxygen generation (∼700 mV) [23]. Figure 4(D) shows the percentage of the INL volume above the stimulation threshold (4.3 mV [9]) during a 4 ms pulse. Previous work has shown that a visual response can be evoked when ∼8% of the targeted volume is above the stimulation threshold [9].

Comparison between Pt and Au pillars demonstrates the effect of sidewall capacitance, altering the potential in electrolyte, especially at the beginning of a 4 ms pulse—figure 4(A). This results in a larger current through the Pt sidewall—figure 4(B) and a lower potential on the Pt sidewall—figure 4(C). In consequence, a greater fraction of current passes through the pillar sidewall and slightly lower fraction of the targeted volume is above the stimulation threshold—figure 4(D).

### 3.2. Fabrication

**Figure 5.**
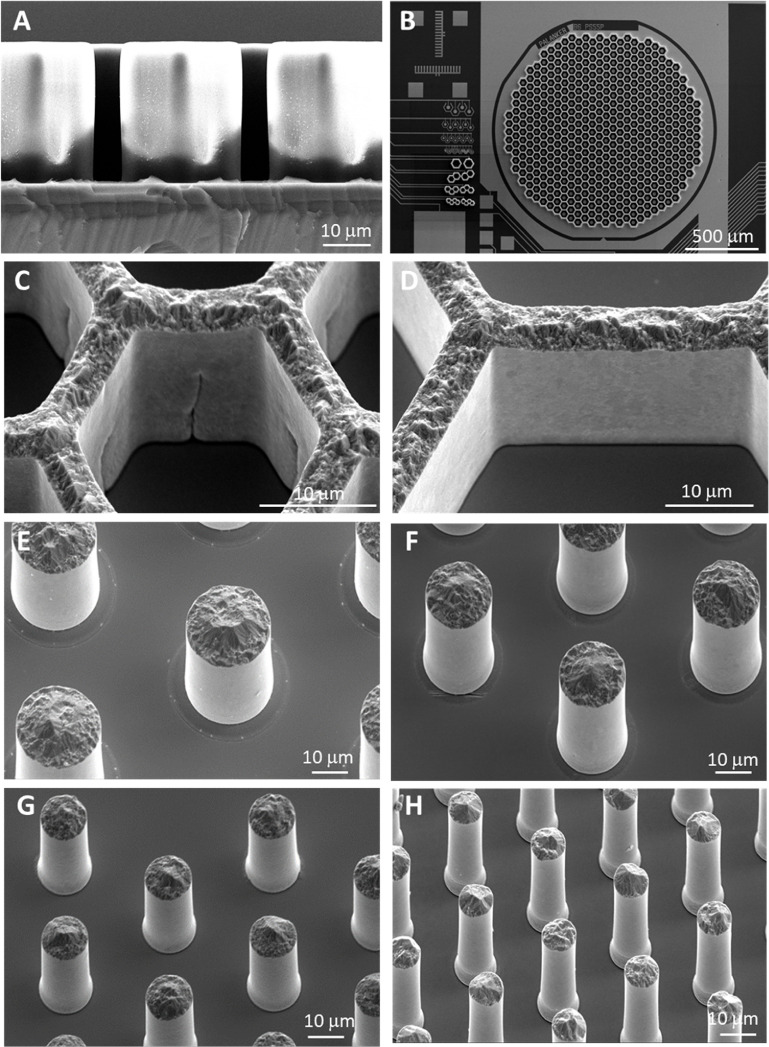
(A) A high-aspect ratio negative photoresist pattern is used to define the electroplated three-dimensional walls. (B) After the wafer scale electroplating process, the photoresist is stripped, leaving 25–35 *μ*m high electroplated structures. (C) and (D) Fabricated structures with smooth sidewalls, of 25 *μ*m in height and 4 *μ*m in width. Shallow surface features are present in the 22 *μ*m pixels as a consequence of reaching the resolution limits for this aspect ratio structure. These surface features do not seem to affect the walls stability, surviving backside grinding and release of individual arrays. (E)–(H) the same fabrication process was adapted to produce pillar electrodes, capable of penetrating past a retinal debris layer, with heights up to 35 *μ*m and widths of 23 *μ*m, 17 *μ*m, 13 *μ*m and 8.5 *μ*m.

Our electroplated honeycombs and pillars are shown in figure 5. A cross section of the photoresist pattern used as an electroplating mask is shown in figure 5(A). For the electroplated honeycomb walls, widths matched the intended dimensions of 4 ± 0.4 *μ*m for 22 and 30 *μ*m pitch pixels, 4.4 ± 0.4 *μ*m for 40 *μ*m pitch pixels and 5.8 ± 0.3 *μ*m for 55 *μ*m pitch pixels. At the base of these walls, we observed a widening of 1.5 ± 0.6 *μ*m. Wall heights across all pixel sizes was 25.0 ± 0.8 *μ*m and these structures, integrated with an underlying device pattern to alignment accuracy of 1 *μ*m (figures 5(B)–(D)). Electroplated pillars had widths of 8.4 ± 0.2 *μ*m, 12.2 ± 0.5 *μ*m, 16.4 ± 0.5 *μ*m and 22.5 ± 0.6 *μ*m for the 22 *μ*m, 30 *μ*m, 40 *μ*m and 55 *μ*m pixel sizes, respectively. At the base, a widening of 0.8 ± 0.6 *μ*m was observed, increasing to 1.8 ± 0.9 *μ*m for 30, 40 and 55 *μ*m pitch pixels. Electroplated pillars of 33.8 ± 1.1 *μ*m in height with diameters matching the size of the active electrodes in the existing photovoltaic arrays [4] are shown in figures 5(E)–(H). These statistics are averaged from 200 pillar/wall structures per pixel size, taken from 5 sites (top, bottom, left, right and center) on 2 wafers.

In principle, iridium oxide (IrOx) could be deposited on top of the gold walls or pillars directly by electroplating (EIROF) [25] or by chemical deposition [26]. However, problems with stability of such films were reported by others [27], and our attempts to reproduce such processes also did not result in a stable coating. Therefore, we applied a more traditional method of IrOx deposition by sputtering (SIROF), which exhibits high capacitance and stability *in-vivo*, and is used in our photovoltaic implants clinically [16] and in animals [6].

**Figure 6.**
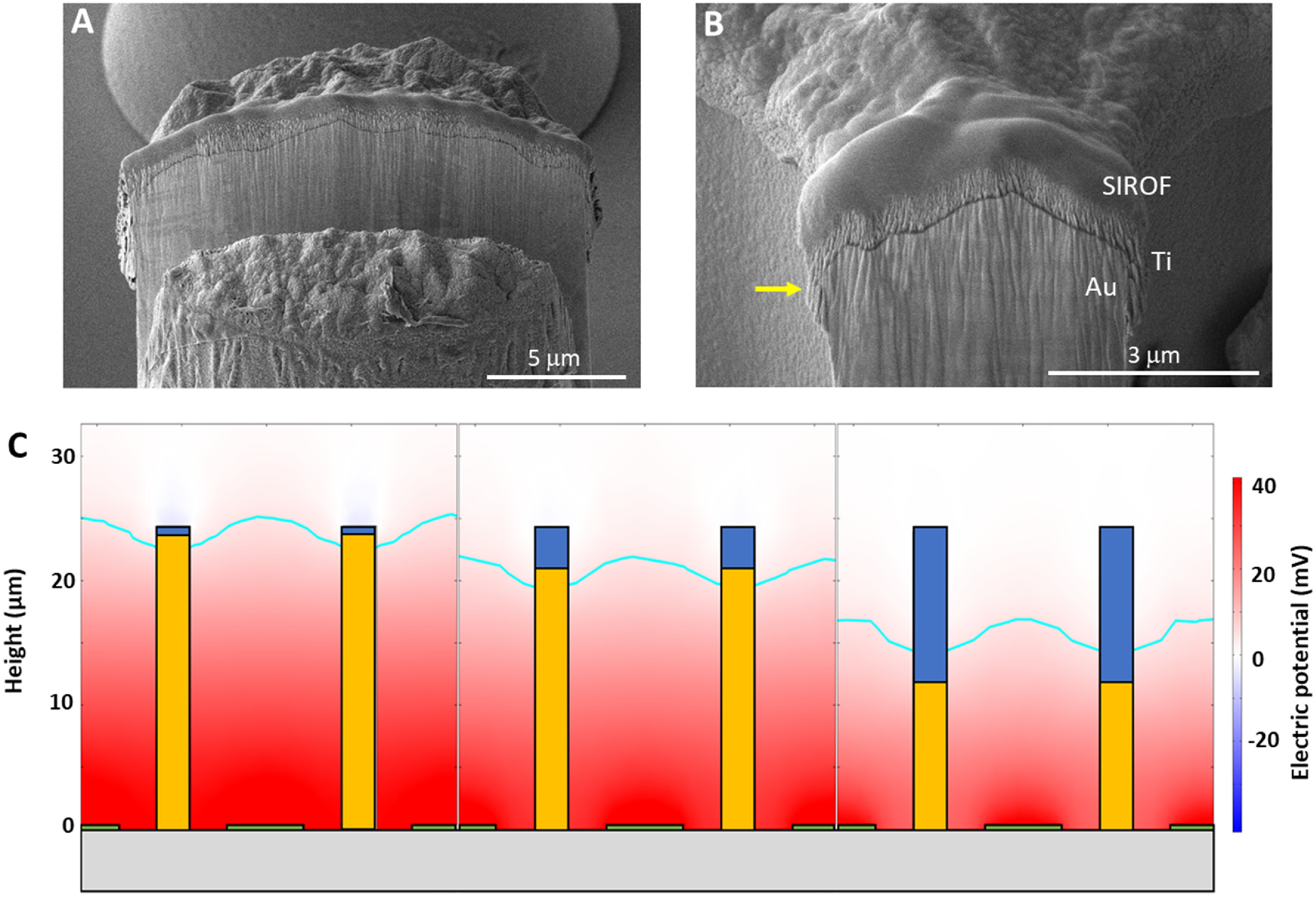
Focused ion beam milling of the SIROF coating on top of electroplated pillars (A) and honeycomb electrodes (B). Deposition of SIROF on top of electroplated gold structures can result in an overhang of a few *μ*m (pointed by an arrow), due to non-uniformities in the lift-off process. (C) Models indicate that an overhang of 4 *μ*m (middle frame) results in a reduction of 7% in the targeted cell volume above the stimulation threshold, whilst an overhang of 12 *μ*m (right frame) would result in a reduction of 17% of this volume.

The top surface of walls and pillars were coated with a 400 nm thick SIROF layer, shown in figure 6(A). Due to variations in height (±0.8 *μ*m) of electroplated structures across a 4 inch wafer and variations in thickness of the lift-off layer, a SIROF overhang of 1–4 *μ*m in height was observed (pointed by the yellow arrow in figure 6(B)). We analyzed the effect of such an overhang on electric field within the honeycombs. As shown in figure 6(C), an overhang of 4 *μ*m decreases the field penetration depth by 7%, compared to the ideal case with a 400 nm SIROF cap. This trend continues, as shown for an extreme overhang covering half of the wall height. Such a large SIROF overhang was not seen in our fabrication results.

**Figure 7.**
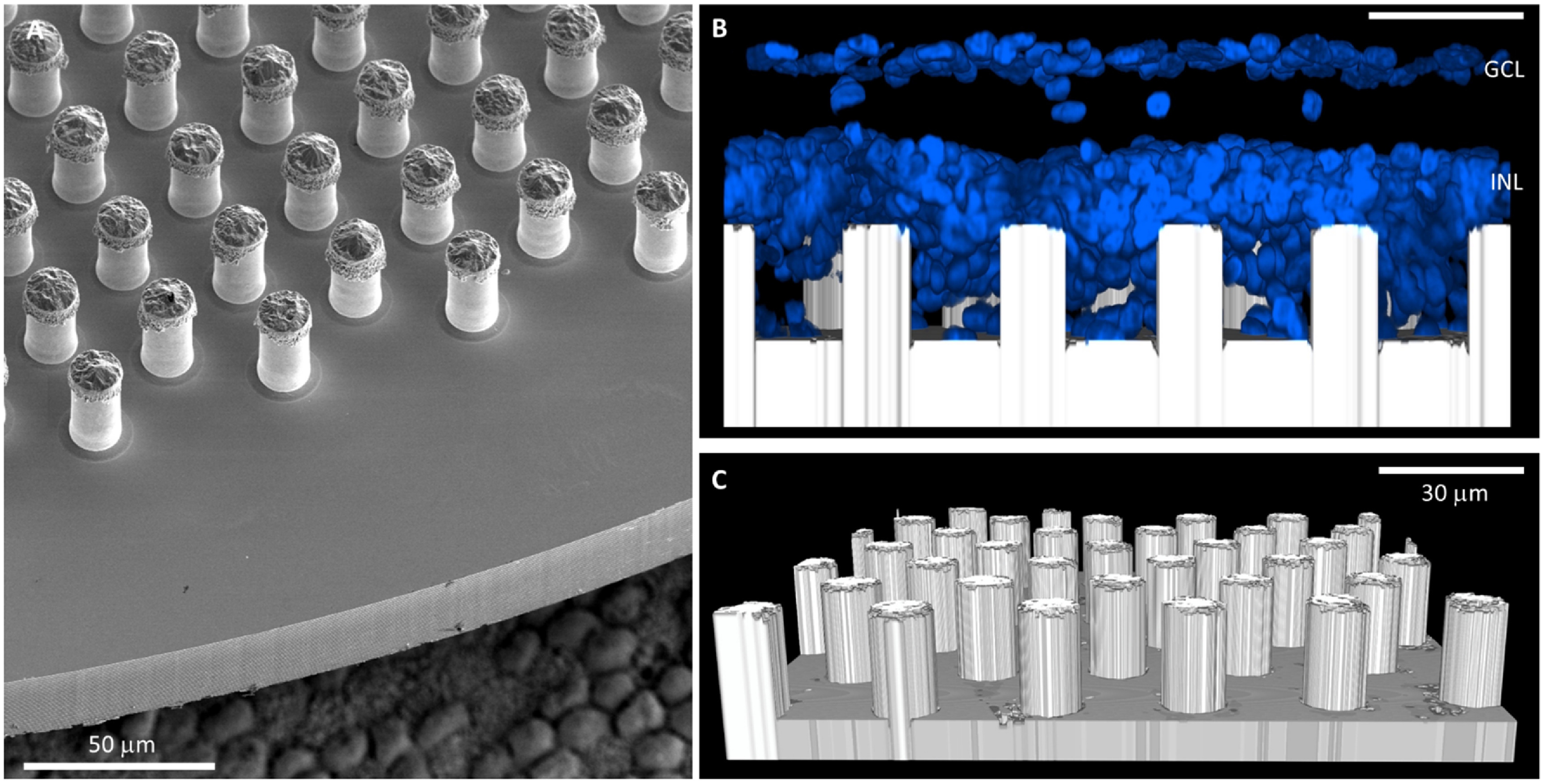
(A) Released array with pillar electrodes on 30 *μ*m pixels, capped with SIROF (implant is placed on top of retinal pigment epithelium to give an idea of scale). (B) Cross section of the explanted structure, integrated with the rat retina. DAPI indicates cell nuclei in blue. (C) Pillars of the implant imaged in the reflection channel of a confocal microscope, without the fluorescence of cells. All the pillars survived the release of individual arrays, implantation and explanation.

We assessed the mechanical stability of these 3D devices by testing their release from the carrier wafer and a subretinal implantation in rats. Since the connected honeycomb walls are much stronger than individual pillars, we focused our effort on evaluating the mechanical stability with pillars. As shown in figure 7(A), all the pillars still stand after the implants release from the carrier wafer. Figures 7(B) and (C) show a cross-section of a confocal image of the device in subretinal space after implantation and explanation 8 d later. Pillars are still standing, and migration of the inner retinal cell into the voids of the array in figure 7(B) indicates feasibility of the tight integration of these 3D structures with the retinal tissue for close proximity to the target neurons. Further details of the retinal integration with 3D implants can be found in [28].

## 4. Discussion

3D electrode structures are essential for high resolution subretinal implants since they improve proximity and enable more focused stimulation of the second-order neurons in the retina. However, fabrication of such structures on semiconductor devices, which rely on planar manufacturing processes, is challenging. One approach to 3D electrodes for retinal prosthesis was developed using an array of crystalline Si pillars, oxidized on the sides and metalized on top, and bonded to the chip [29]. Such process was demonstrated for 100 *μ*m pixels, but it is difficult to scale it down to much smaller pixel pitch, such as 20 *μ*m. Another approach could be based on 3D polymer structures, which can be formed by 2-photon laser lithography and subsequently metalized for conduction [30–32]. However, polymer features (pillars or walls) of high aspect ratio tend to deform over time, leading to cracking of the metal coating.

Here, we show how 3D devices can be produced using conventional lithographic techniques coupled to a gold electroplating process that enables mechanically robust high-aspect ratio structures in the form of either honeycomb walls or pillars. The process is compatible with the post-processing of wafer-level devices and is robust enough to withstand the mechanical thinning of the wafers, release of the individual devices and implantation in rats. Furthermore, the 3D electrode structures integrate well with the retinal tissue, with histological results showing migration of the inner retinal neurons into the voids between electrodes, promoting close proximity between the stimulation electrodes and cells.

While we show feasibility of fabricating 35 *μ*m tall pillars of 8.5 *μ*m in diameter, or 25 *μ*m high honeycomb walls of 4 *μ*m in width, it is important to note that the KMPR photoresist layer, used to define the electroplated structures, needs significant process optimization for the highest aspect ratio structures. Furthermore, optimization of the UV exposure doses, photolithography mask design and oxygen plasma clean cycles for the smallest features, lead to over-exposure of larger features, resulting in widening of the base of larger electroplated features. The implication is that it would be difficult to fabricate 3D-electrode interfaces with very different sizes (and aspect ratio of height to width) on the same wafer. In a manufacturing setting, this challenge can be addressed by having arrays of devices with only one pixel size per wafer.

Narrower wells, such as the 25 *μ*m deep honeycombs on 22 *μ*m pixels in figure 5(C), exhibit surface features. During the oxygen plasma etch, used to clear out the photoresist pattern, residue can be left in these narrower features, which are then imprinted on the electroplated walls. The mechanical stability is unaffected, as the devices were successfully thinned and released with no losses.

An important consequence of this fabrication process is the exposed metallic sidewalls of the 3D structures. Ideally these would be insulated by a dielectric thin film, however, this is challenging on such high-aspect ratio 3D structures and the non-conformal coating of most deposition techniques. Modeling indicates that smooth (low capacitance) Au sidewalls do not provide significant charge transfer, which is dominated by the (high capacitance) SIROF electrodes on the top surface of the 3D structures. The Au sidewalls are charged predominantly during a submillisecond time window on the rising and falling edges of the stimulation pulse. The magnitude depends upon the double layer capacitance, with 5%–25% charge loss over the likely range of C_DL_ values (14–56 *μ*F cm^−2^). On pillar active electrodes, the potential transients required to deliver these stimulation pulses are positive and quite low (<100 mV). On honeycomb return electrodes, these transients are negative, and even lower (about −40 mV). Since the gold walls have much lower capacitance than the connected SIROF caps, their equilibrium potential will follow that of SIROF, which is about +200 mV vs. Ag/AgCl [33]. Therefore, negative voltage transients below 500 mV in amplitude should not exceed the threshold for oxygen reduction or H_2_O_2_ evolution (−300 mV), and positive transients below 500 mV will not result in O_2_ generation (+700 mV) [23]. Electrode capacitance *in-vivo* might be lower than *in-vitro*: e.g. 2–3 times for SIROF [34], and 3–5 times for Pt [35]. For the same charge injection, smaller capacitance will result in a larger voltage drop across the electrode-electrolyte interface. However, with a single Si photodiode per pixel, the maximum photovoltage of about 500 mV, divided between the anode, cathode and ohmic drop in the medium, is unlikely to exceed the thresholds of these electrochemical reactions at clinical levels of irradiance (<3.5 mW mm^−2^). Therefore, as long as the potential at the Au interface is kept away from the redox reaction levels, then the low capacitance of these exposed side walls obviates the need for their electrical insulation, greatly simplifying the microfabrication process.

Although the models indicate that the surface potentials and charge densities on Au surfaces are below the commonly quoted values for detrimental reactions *in vivo*, a more common electrode material is platinum. Pt electrodes are a clinical standard in very successful long-term neural implants, such as deep brain stimulators and cochlear implants [36]. This makes Pt an attractive material for 3D electrodes, however, there is a caveat. Higher *C*_DL_ of Pt (100 *μ*C cm^−2^) compared to Au means more charge is driven across the exposed walls during stimulation, reducing the current delivered via the SIROF cap to the target cells. Also, stronger catalytic ability of Pt results in lower threshold for the onset of oxygen reduction (100 mV) [23]). Our models indicate that more than half of the targeted cells can still be safely driven above the stimulation threshold (4.3 mV) using modest irradiance values (∼1 mW mm^−2^), when the potential on sidewalls does not exceed the onset of oxygen reduction on honeycomb walls. However, if stronger stimulation is applied (e.g. 3 mW mm^−2^ used with the PRIMA implants in clinics), it may result in higher voltages, exceeding the threshold of irreversible electrochemical reactions. To prevent them, atomic layer deposition coatings could be introduced. Such insulating coatings on sidewalls could eliminate the possibility of any reactions and further concentrate the electrical current through the SIROF caps.

These 3D electrode structures electroplated on top of the planar subretinal arrays hold promise for either shaping the electric field vertically (honeycombs) or raising the stimulating electrode to the target neuronal layer (pillars), both of which improve the efficiency of retinal stimulation and will help facilitate the high-density neuromodulation.

## Data Availability

All data that support the findings of this study are included within the article (and any supplementary files).
